# Classification of Mental Stress from Wearable Physiological Sensors Using Image-Encoding-Based Deep Neural Network

**DOI:** 10.3390/bios12121153

**Published:** 2022-12-09

**Authors:** Sayandeep Ghosh, SeongKi Kim, Muhammad Fazal Ijaz, Pawan Kumar Singh, Mufti Mahmud

**Affiliations:** 1 Department of Instrumentation and Electronics Engineering, Jadavpur University, Jadavpur University Second Campus, Plot No. 8, Salt Lake Bypass, LB Block, Sector III, Kolkata 700106, West Bengal, India; 2 National Centre of Excellence in Software, Sangmyung University, Seoul 03016, Republic of Korea; 3 Department of Intelligent Mechatronics Engineering, Sejong University, Seoul 05006, Republic of Korea; 4 Department of Information Technology, Jadavpur University, Jadavpur University Second Campus, Plot No. 8, Salt Lake Bypass, LB Block, Sector III, Kolkata 700106, West Bengal, India; 5 School of Science and Technology, Nottingham Trent University, Clifton, Nottingham NG11 8NS, UK; 6 Department of Computer Science, Nottingham Trent University, Clifton, Nottingham NG11 8NS, UK; 7 Medical Technologies Innovation Facility, Nottingham Trent University, Nottingham NG11 8NS, UK; 8 Computing and Informatics Research Centre, Nottingham Trent University, Nottingham NG11 8NS, UK

**Keywords:** stress detection, Gramian Angular Field, deep neural network, WESAD dataset, SWELL dataset

## Abstract

The human body is designed to experience stress and react to it, and experiencing challenges causes our body to produce physical and mental responses and also helps our body to adjust to new situations. However, stress becomes a problem when it continues to remain without a period of relaxation or relief. When a person has long-term stress, continued activation of the stress response causes wear and tear on the body. Chronic stress results in cancer, cardiovascular disease, depression, and diabetes, and thus is deeply detrimental to our health. Previous researchers have performed a lot of work regarding mental stress, using mainly machine-learning-based approaches. However, most of the methods have used raw, unprocessed data, which cause more errors and thereby affect the overall model performance. Moreover, corrupt data values are very common, especially for wearable sensor datasets, which may also lead to poor performance in this regard. This paper introduces a deep-learning-based method for mental stress detection by encoding time series raw data into Gramian Angular Field images, which results in promising accuracy while detecting the stress levels of an individual. The experiment has been conducted on two standard benchmark datasets, namely WESAD (wearable stress and affect detection) and SWELL. During the studies, testing accuracies of 94.8% and 99.39% are achieved for the WESAD and SWELL datasets, respectively. For the WESAD dataset, chest data are taken for the experiment, including the data of sensor modalities such as three-axis acceleration (ACC), electrocardiogram (ECG), body temperature (TEMP), respiration (RESP), etc.

## 1. Introduction

About 280 million people suffer from depression every year and very few of them obtain proper treatment on time [1]. Therefore, it is very important to detect human stress so that more people become aware of their situation and obtain their treatment as soon as possible. Stress can spoil someone’s quality of life in many ways which are difficult to imagine [2]. Human beings are well adapted to stress in small doses, but when that stress is long-term, it can have some serious impacts on our bodies as well [3]. It also causes the muscles in our body to be in a constant state of guardedness. Taut and tense muscles for long periods cause other portions of the body to react and even promote stress-related disorders. Stress can also cause respiratory symptoms such as shortness of breath and rapid breathing, as the airway between the nose and the lungs constricts [4]. 

The main source of stress response in human beings comes from the sympathetic nervous system (SNS), which mainly carries physiological, psychological, and behavioural symptoms [5]. Psychological responses are mainly anger, irritation, anxiety, or depression. From a physiological perspective, when SNS activity increases it changes the hormonal levels of the body and provokes reactions like sweat production, increased heart rate, and muscle activation [6]. The muscles mainly control the respiratory system and vocal tract, so when the muscles change it causes our speech characteristics to change as well. In addition, skin temperature decreases [7] along with hands and feet temperature, and heart rate variability (HRV) [8] decreases along with a change in pupil diameter [9]. In the case of the behavioural point of view, eye gaze and blink rate variations in addition to changes in facial expressions or head movement are affected in a lot of ways [10]. Carrying out a continuous process of tracking stress manually is far from reality. Moreover, carrying out methods of psychological questionnaires is nearly impossible for the detection of stress. This is where automatic stress recognition comes into play. Hormone levels also play an active role in stress. The stress response causes endocrine and immune systems to change by releasing adrenaline and cortisol hormones from the adrenal cortex and adrenal medulla, respectively [11]. On the other hand, in the case of automatic stress detection, we measure some of the most important factors affecting human stress or possible for recognizing stress more accurately, which include the bio-signals such as ECG, EDA, signals, etc., and reduce a lot of manual effort in parallel. There are several traditional methods of detecting stress, such as interviewing the individual by asking stress-related questions or observing the reactions of people who are stressed giving different facial expressions, i.e., their blinking rate, pupils, or eyebrow rate.

**Figure 1:**
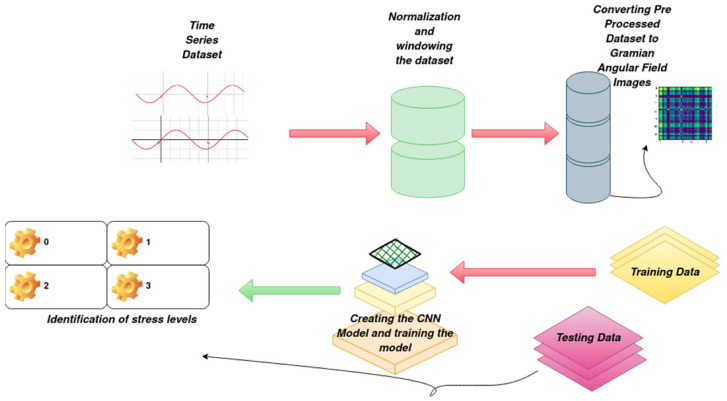
Illustration of the whole pipeline of our proposed image-encoding-based deep neural network for mental stress detection from wearable physiological sensors.

Some relevant contributions of the proposed work include the following:

- The present work encodes a multivariate time series dataset to time series images which resulted in promising accuracies achieved in both training as well as testing phases.
- The work properly groups the multivariate time series dataset which is being experimented on for the first time and converts it to Gramian Angular Field (GAF) images successfully before training the normalized data with the help of a convolutional neural network (CNN). An overview of our proposed pipeline for mental stress detection is illustrated in Figure 1.
- The proposed image-encoding-based deep neural network model is tested on two standard benchmark stress recognition datasets, namely WESAD [12] and SWELL [13]. This resulted in better classification accuracies which proved that the model is capable of showing good performances on any time series dataset.

## 2. Literature Review

A lot of research has been conducted in the past using machine learning techniques for stress detection. In the work by Bobade et al. [4], stress detection was achieved on the publicly available WESAD dataset using data from sensor modalities such as ACC, ECG, EDA, etc., for both binary and non-binary classification. In the case of non-binary classification, three-class classification was conducted using machine learning techniques such as K Nearest Neighbour, Linear Discriminant Analysis, Random Forest, Decision tree, AdaBoost and Kernel Support Vector Machines. During the study, an accuracy of about 81.65% was achieved for the three-class classification. However, the related work was implemented on an old structured WESAD dataset consisting of three stress classes excluding the meditation class which has been updated recently. The second work conducted by Souza et al. [14] proposes a new model called MoStress which depends on a sequence model for stress classification. It pre-processes the physiological data collected from wearable devices through a novel pipeline using a recurrent neural network (RNN). Although the paper claims that the result is nearly close to other proposed works, they used a simpler model. Some different approaches have been applied as well by Rashid et al. [15], where they applied motion which determines the context of the system while also learning to adjust the fused sensors whenever required. Some research work has been conducted on stress detection using deep learning models. Sah et al. [16] introduced the CNN model for stress detection by using the data of only one sensor modality. Ghosh et al. [17] worked on another method for mental stress detection using two physiological signals. They proposed a statistical feature extraction taken from a 10 s segment which is performed by wavelet packet decomposition, which also follows a multi-class Random Forest classifier. Chatterjee et al. [18] proposed a lightweight deep neural network which detects mental stress using physiological signals. They took ECG, Galvanic Skin Response (GSR), skin temperature, and EMG signals using a wearable device. An accuracy of 90% was achieved by them for the three class classifications.

Much research work involving stress detection has been performed on the SWELL dataset as well. Sharma et al. [19] conducted stress detection using machine learning classifiers along with the Internet of Things Environment. With the popularity of smartwatches, the work proposed that the data collected from the watches can be trained using machine learning algorithms and can be shared with experts for the best possible recommendations regarding health. This also includes the study of recommender systems using IoT and the cloud, which achieved an accuracy of 98%. Another work was conducted by Ragav et al. [20] regarding Bayesian active learning for wearable stress and affect detection. This work handled data using the ground truthing technique or active learning. This work introduced a Bayesian neural network technique along with Monte Carlo Dropout to predict model uncertainties using approximation, which achieved an accuracy of 90.38%. The authors of [21] proposed an artificial neural network to detect and classify stress with an accuracy of 78% and an error rate of about 22%, respectively. Koldijk et al. [22] proposed and developed automatic classifiers to detect stress-related mental states, especially in working conditions using computer logging, facial expressions, as well as physiology. They mainly addressed two methods of applied machine learning challenges. Firstly, they detected work stress using unobtrusive sensors followed by taking individual differences into account. They also found that it is better to predict variable mental effort using sensor data than perceived stress. Nkurikiyeyezu et al. [23] worked on the SWELL dataset in their work, addressing the two most important questions, among which one is related to heart rate variability and another to distinguishing between stressful and non-stressful situations in an office-related environment. They achieved an accuracy of 99.25% related to stress predictions. They mainly used machine learning methods, which were trained on 10-fold cross-validation of the training dataset where each fold was used to train on random forest classifiers using the remaining 9 folds. After testing various machine learning classifiers, they settled with the Decision Jungle (Shotton et al. [24]). They tend to generalize better with less memory consumption.

### Time Series Images

A time series represents a series of time-based orders. It is basically a sequence of various data points that occurred in a successive order for a given time. There are many applications of time series analysis in different fields, ranging from weather forecasting to financial purposes to signal processing and many more. The specific experiment focuses on classification, although regression is also possible with time series analysis and using time series images as well. With the recent developments of computer vision, time series images have also become popular as well. There are several ways to encode time series datasets into images. One such example is GAF. A GAF is an image obtained from a time series, representing some kind of temporal correlation between each pair of values from the time series.

The mathematics of the GAF is intrinsically linked to the inner product and the corresponding Gram matrix. The inner product is an operation between two vectors, which measures their similarity [25]. Let us consider there are two vectors x and y. The inner product between them is the dot product which can be written as the following:

(1) $$\langle x,y\rangle =x1\cdot y1+x\cdot y2\;$$

which can be further simplified as follows:

(2) $$\langle x,y\rangle =\left|\left|x\right|\right|\cdot \;\left|\left|y\right|\right|\cdot \;cos\left(\theta \right)$$

Therefore, the inner product between them can be characterized by the angular difference cosθ. The resulting value lies between $\left[-1,1\right]$. The matrix of a set of *n* such vectors defined by the dot product of every couple of vectors is called the Gram matrix [26]. The Gram determinant or Gramian is the determinant of the Gram matrix:

(3) $$\left|G\;\left(\left\{x1,\;x2,.xn\right\}\right)\right|=\left|\begin{array}{ccc} \langle x1,x1\rangle & \langle x1,x2\rangle \;\ldots & \langle x1,xn\rangle \\ \langle x2,x1\rangle & \langle x2,x2\rangle \;\ldots & \langle x2,xn\rangle \\ \langle x3,x1\rangle & \langle x3,x2\rangle \;\ldots & \langle x3,xn\rangle \\ \langle x4,x1\rangle & \langle x4,x2\rangle \;\ldots & \langle x4,xn\rangle \\ \ldots & \ldots & \ldots \\ \langle xn,x1\rangle & \langle xn,x2\rangle \ldots & \langle xn,xn\rangle \end{array}\right|$$

**Figure 2:**
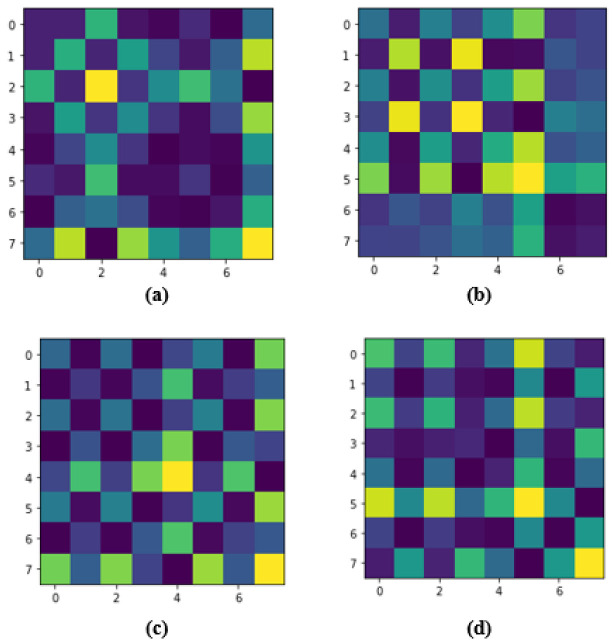
Illustration of random GAF images transformed from the normalized WESAD dataset: (**a**) level 0 (Meditation), (**b**) level 1 (Baseline), (**c**) level 2 (Stress) (**d**) level 3 (Amusement).

**Figure 3:**
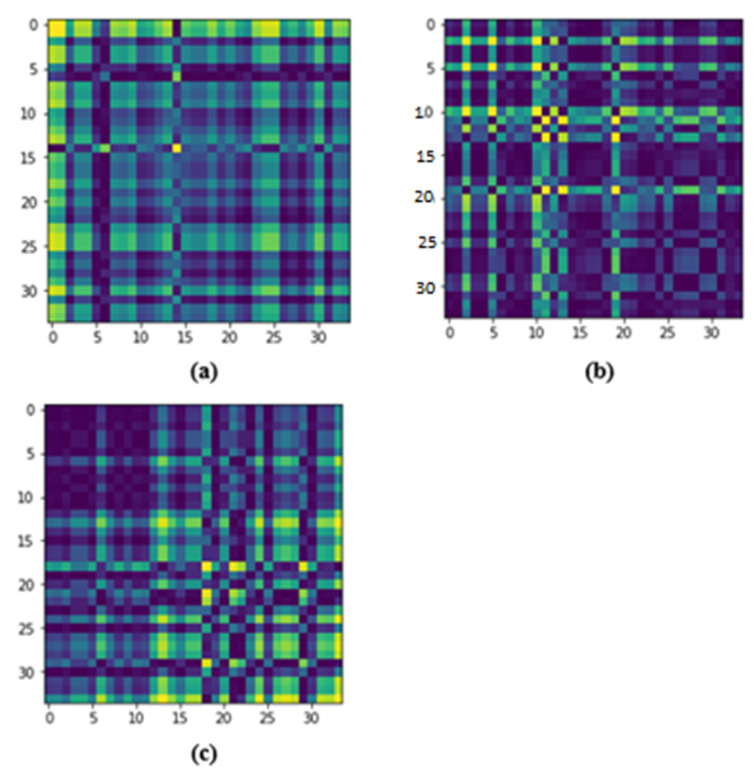
Illustration of random GAF images transformed from the normalized SWELL dataset: (**a**) level 0 (No Stress), (**b**) level 1 (Time Pressure), (**c**) level 2 (Interruption).

The specialty of this matrix is that the time dimension is encoded into the geometry of the matrix. As the position moves from the top-left to the bottom-right, time increases as well. Since the time series is scaled, we can compute pairwise dot products and store them in the Gram matrix. Time series are also cosines, so the Gram matrix follows a Gaussian distribution as well. The resulting image is also noisy as a result of this. If we extract the dataset in the form of data frames, then each row in the data frame will produce one Gram matrix, which is shown in Figure 2 for the WESAD dataset where the GAF image for each identification label is shown. Similar work has been performed for the SWELL dataset, which is shown in Figure 3, but the number of stress labels is three as compared to the WESAD dataset which has four.

## 3. Datasets Used

For the experiment, two time series datasets were tried and tested by encoding them to time series images and normalizing them before passing the images to a convolutional neural network. The first dataset is the publicly available WESAD. This multimodal dataset features physiological and motion data, recorded from both a wrist- and a chest-worn device, of 15 subjects during a lab study. The following experiment was conducted on the chest data. Therefore, the following sensor modalities which are particularly related to the chest, three-axis acceleration (ACC), electrocardiogram (ECG), body temperature (TEMP), respiration (RESP), electrodermal activity (EDA), electromyogram (EMG), were considered and extracted from the dataset.

The second time series dataset called the SWELL dataset [2] was collected by researchers at the Institute for Computing and Information Sciences at Radboud University. The experiment (related to the SWELL dataset) was conducted on 25 people performing normal work related to the office. Various data were collected including computer logging, facial expression, body postures, ECG signal, and skin conductance, especially when the people were receiving unexpected email interruptions and pressure to complete their work on time.

## 4. Proposed Methodology

The main aim of the research paper is to propose a new and promising technique for stress detection using CNN and encoding the multivariate time series dataset to GAF images after correctly pre-processing the dataset followed by necessary transformation as well as normalization. In the case of the WESAD dataset, the chest data of an individual among different subjects for which the data have been recorded were taken and extracted and converted to data frames, keeping chest sensor keys as the columns, and the labels were taken separately from the data frames. The labels consist of the stress level ranging from ‘0’ to ‘3’. For the SWELL dataset, the data of computer logging, facial expression, body postures, ECG signal, and skin conductance of an individual among 25 different subjects were taken, extracted, and converted to data frames, and labels were taken separately. The labels are the stress identification labels ranging from ‘0’ (No Stress) to ‘2’ (Maximum Stress).

### 4.1. Extracting Dataset and Normalization

The data are grouped based on labels with data whose stress levels are ‘0’, and are kept together as well for stress levels ‘1’, ‘2’, and ‘3’. The data are arranged on the basis explained before and after the last 10,000 data points are taken from each group for 4-class classification using a CNN model after encoding them to GAF Images. The data are the normalized first quantile. Numerical input variables may have a highly skewed or non-standard distribution, which may be caused by outliers in the data, multimodal distributions, or highly exponential distributions. Many machine learning algorithms perform better when numerical input variables and output variables in the case of regression have a standard probability distribution, especially a Gaussian (normal) or uniform distribution. This is why quantile normalization is so useful. Firstly, we sort each column independently. The average of each computation is computed where each row is in ascending order. Finally, the row average values which are also the mean quantiles are replaced with the raw data in the right order.

### 4.2. Encoding Dataset to Time Series Images

**Figure 4:**
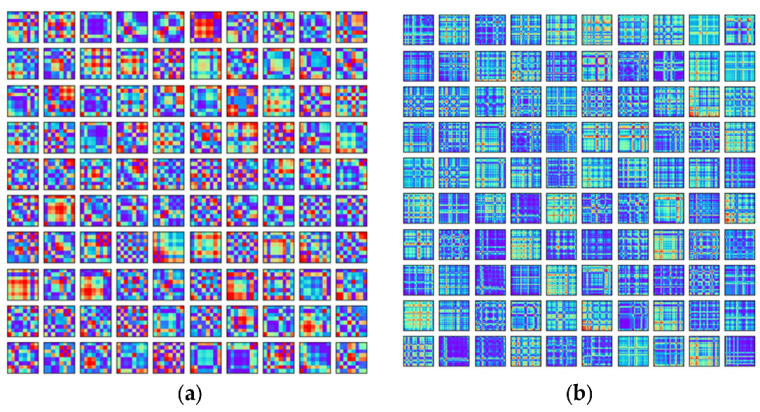
Illustration of the GAF image for 100 rows of normalized (**a**) WESAD and (**b**) SWELL datasets in the form of a 10 × 10 square matrix.

After extraction and normalization, the whole dataset (which is the part of the actual data which we took for the training process) was converted to GAF images. Figure 4 illustrates the *GAF* image for 100 rows of normalized WESAD and SWELL datasets in the form of a 10 × 10 square matrix. From Figure 4, it seems like the image encoding for the SWELL dataset is different from that encoded for the WESAD dataset, since the colour instance map has been changed to a rainbow in this case.

### 4.3. Creating the CNN Model

Further normalization is required for passing the data to the CNN. The time series matrix computed for all the rows taken for the experiment was reshaped and all the labels were converted to a class matrix of binary digits. The CNN model was finally created for training the data after splitting them into training and testing data with a ratio of 3:2.

**Figure 5:**
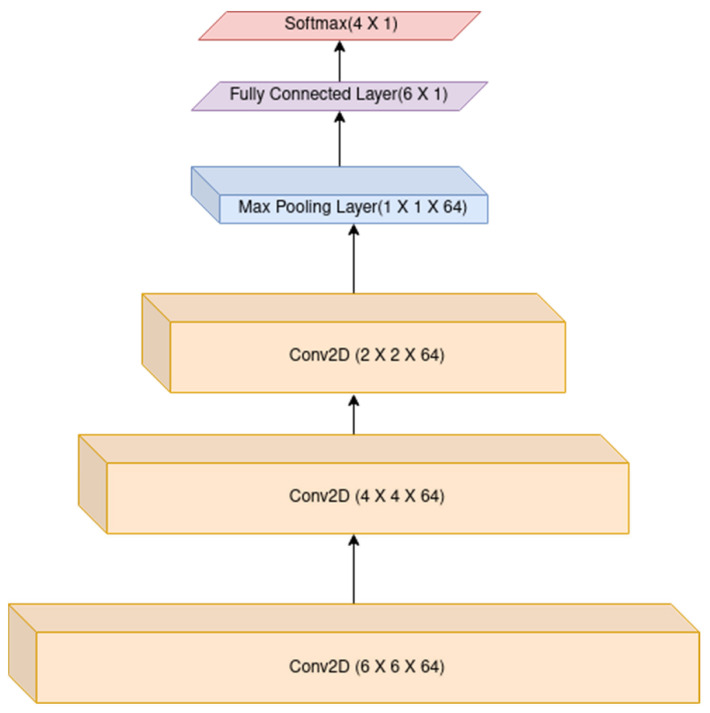
Illustration of the proposed custom-built CNN architecture with 4 output classes for the WESAD dataset and 3 in the case of the SWELL dataset.

Figure 5 describes our proposed custom-built CNN model which was formed with 3 convolutional layers with an activation function set to ReLU (Rectified Linear Activation function) with 3 × 3 kernel size and 64 filters followed by the application of the Batch Normalization technique. It normalizes the contribution to a layer for every mini-batch of data. A detailed version of the model is also shown in Table 1, describing the layers of the custom-built CNN model. After the convolutional layers, a pooling layer is present for selecting the maximum values in the receptive fields of the input. After saving the indices it produces a summarized output volume. Finally, two dense layers were created with an activation function set to Softmax for multi-class classification purposes, which is a 4-class classification in the case of the WESAD dataset and a 3-class classification in the case of the SWELL dataset.

It is to be noted from Table 1 that in the case of the SWELL dataset, the output layer is 3, which is 4 in the case of the WESAD dataset for final stress detection.

## 5. Results and Discussion

The following experiment was performed using DELL Laptop Inspiron 15 5518 with 16 GB memory and 8 GB Random Access Memory (RAM) with an 11th Gen Intel Core processor. An Ubuntu 22.04 1 LTS 64-bit operating system was used and the entire source code for this experiment was written with the help of a jupyter notebook. Before calculating training and testing accuracies attained by the proposed image-encoding-based deep neural network, we also take note of the four evaluation metrices, accuracy, precision, recall, and F1 score, used in the present work. They are defined below as follows:

Accuracy of a model is defined as the fraction of the total number of correct predictions divided by the total number of predictions being made by our model. It helps in evaluating the performance of the model being used for classification in this regard.

(4) $$Accruacy=\frac{Total\;Number\;of\;Correct\;Predictions}{Total\;Number\;of\;Predictions}\times 100\%$$

Precision detects the correctness of the proportion of identifications in a model [27].

(5) Precision=xpxp+yp

where $x_{p}$ and $y_{p}$ are the numbers of true positives, and false positives are classified by the model.

Recall detects the correctness of the proportion of actual positives being correctly identified by the model [27].

(6) Recall=xpxp+yn

where $y_{n}$ is the number false negatives being classified by the model.

The F1 score is a measure of model accuracy on a dataset which is also used to evaluate binary classification systems [28].

It can be represented by the formula

(7) $$F1\;score=\frac{2}{\frac{1}{Precision}+\frac{1}{Recall}}$$

### 5.1. WESAD Dataset

**Figure 6:**
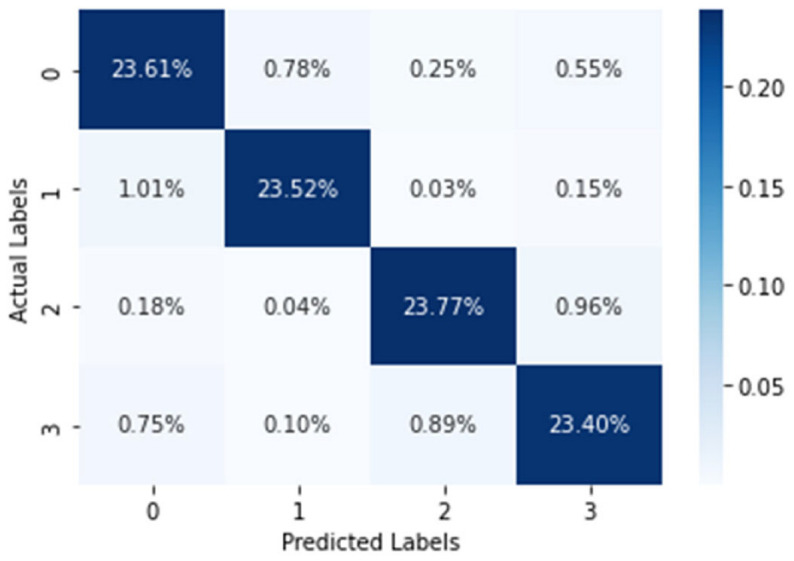
Confusion matrix generated by the proposed image-encoding-based deep neural network for the WESAD dataset where the percentage of data are being displayed in each quadrant.

**Figure 7:**
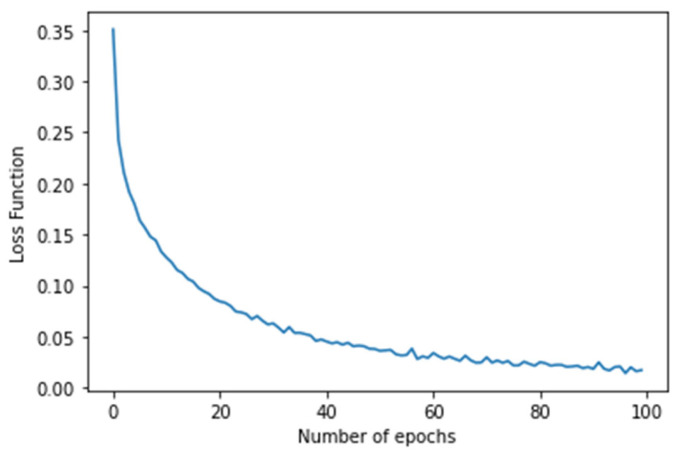
Graph showing the plot of the loss function with several epochs in the *x*-axis and its corresponding losses in the *y*-axis.

**Figure 8:**
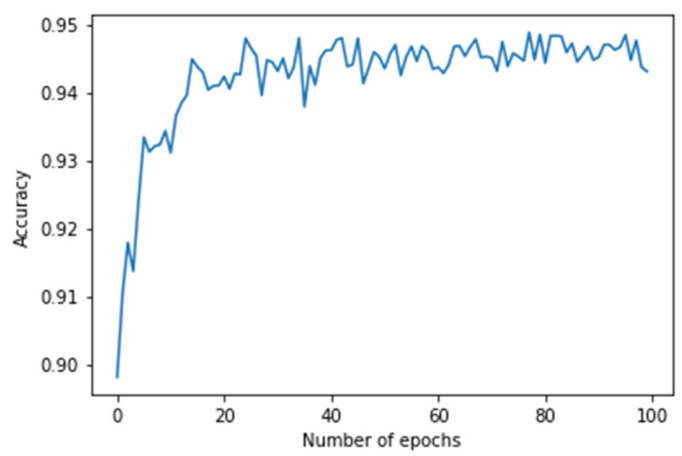
Graph showing the accuracy vs. the number of epochs with the *X*-axis as epochs and the *Y*-axis as its corresponding accuracies.

In the case of the WESAD dataset, the experiment was conducted to predict the stress level of an individual ranging from 0 (Baseline) to 3 (Amusement). After training for around 100 epochs, a promising training accuracy of 99.48% and testing accuracy of 94.77% was achieved. The confusion matrix produced by the proposed image-encoding-based deep neural network model for the WESAD dataset is shown in Figure 6, where the *X*-axis represents the predicted labels and the *Y*-axis shows the actual labels of the data. Table 2 shows the stress-wise performance of the proposed model for the WESAD dataset, which displays the accuracy, precision, recall, and F1 score for each stress identification label along with the average of all the individual stress-wise performances. Figure 7 and Figure 8 illustrate the variation in the loss function and classification accuracy with respect to the number of epochs, respectively. It can be examined from Figure 7 and Figure 8 that the graphs plotted for loss function decrease drastically, whereas the classification accuracy increases as the model is trained for a greater number of epochs. The results also confirm that encoding a multivariate time series dataset to its corresponding image provides more enhanced accuracy as compared to other related works without the application of encoding time series images.

### 5.2. SWELL Dataset

**Figure 9:**
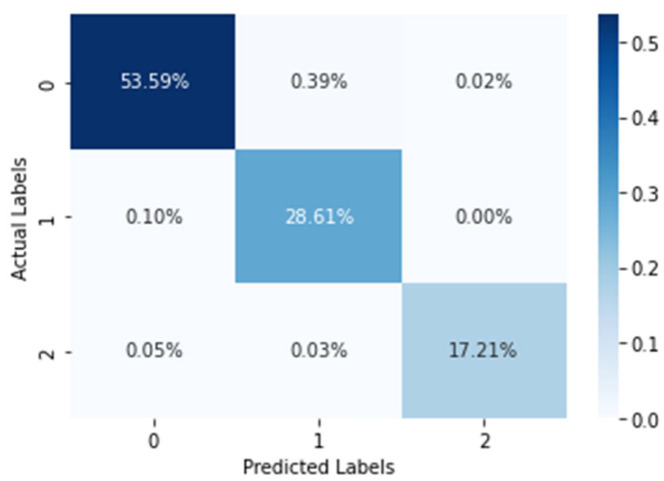
Confusion matrix generated by the proposed image-encoding-based deep neural network for the SWELL dataset where the percentage of data are being represented in each quadrant.

For further clarification, another dataset called the SWELL dataset was also extracted, normalized, and encoded to GAF images following a similar procedure as was performed in the case of the WESAD dataset trained with the help of the same model, which also produced a training accuracy of 99.49% and testing accuracy of over 99.39%. The results are more promising as compared to the results obtained from any other related works which involve converting the time series to a spectrogram. This is disadvantageous, since in a spectrogram it matters where an effect appears, in contrast to CNNs where it is assumed that a feature is of the same kind irrespective of its location. The confusion matrix produced by the proposed image-encoding-based deep neural network model for the WESAD dataset is shown in Figure 9, where the *X*-axis represents the predicted labels and the *Y*-axis shows the actual labels of the data. Table 3 shows the stress-wise performance of the proposed model for the SWELL dataset, which displays the accuracy, precision, recall and F1 score for each stress identification label along with the average of all the individual stress-wise performances. It can be observed form Table 3 that the F1 score for the SWELL dataset is found to be more than that of the WESAD dataset. Since the length of the SWELL dataset is considerably small with respect to the WESAD dataset, the proposed deep neural network is performing better in this regard. In the case of a larger dataset, the time taken for the collection of data is more, which also causes the battery of the RespiBAN device being used for data collection to drain out more as compared to a smaller dataset. This in turn affects the classification accuracy with which the data are being collected.

### 5.3. Summarization of Results

After performing the experiment on the two benchmark datasets and calculating individual class-wise accuracy as well as their F1 score, precision, and recall, we took the average of all the classes and displayed them in Table 4. It can be seen from Table 4 that the proposed image-encoding-based deep neural network produces classification accuracies of 94.77% and 99.39% for the WESAD and SWELL datasets, respectively. For the SWELL dataset, the length of data is small compared to the WESAD dataset, and it took a significantly smaller number of epochs to train the model, for which the plot of loss function versus epoch size and accuracy versus epoch size are not necessary in that case, since the number of epochs would be negligible as compared to the WESAD dataset.

### 5.4. Comparison with Existing Stress Recognition Models

Table 5 and Table 6 show the comparison of the classification accuracy of our proposed work with respect to the accuracy obtained in previous works for WESAD and SWELL datasets, respectively. It is observed from Table 5 and Table 6 that the overall mental classification performance is found to be very promising as compared to previous research works being conducting on both the datasets for the multi-stress classification problem. The work performed in the year 2021 on the WESAD dataset by Sah et al. [16] achieved a promising accuracy of about 92.85% using CNN. Other works using the RNN model for stress classification include that by Melchiades et al. [14] in 2022, which achieved an accuracy of 86% for the WESAD dataset, whereas Bobade et al. [4] describe machine learning techniques for stress detection, achieving an accuracy of 84.32% in the year 2020. It is to be noted that all of the abovementioned works have reported their accuracies for multi-class classification. There are also some promising research works which have been conducted for the SWELL dataset, including that by Sharma et al. [19], achieving an accuracy of 98% in 2019 using the Internet of Things (IoT) Environment, and Ragav et al. [20], attaining an accuracy of 90.38% using Bayesian neural network in 2020. The authors of [29] used machine learning techniques for identifying stress, which was very promising, and the authors of [30] successfully used a tiled convolutional neural network after encoding time series images for stress recognition. Hatami et al. [31], Chen et al. [32], and Xu et al. [33] also conducted experiments on deep convolutional neural networks in their experiments with the help of time series images and achieved promising results in their proposed work. Bragin et al. [34] successfully revealed the usage of GAF conversion of EEG signals in their experiments as well. The work conducted by Walambe et al. [35] used a multimodal framework for stress detection and achieved a promising accuracy of 96.09% for the SWELL dataset in 2021. Han et al. [36] successfully used the application of GAF images in their experiment to introduce a new Bearing Fault Diagnosis Method, which is not related to stress recognition, but showed a promising approach to using GAF images and their implementation which certainly helped us to understand more about time series images in this regard. The authors of [37] successfully introduced a hierarchical deep neural network for mental stress state detection using IoT-based biomarkers. The authors of [38,39] also performed promising work on developing deep neural networks for stress recognition by using data being collected from wearable sensors. The authors of [40] identified biomarkers for accurate detection of stress in their research work. Iqbal et al. [41] successfully analysed biophysiological responses of stress for wearable sensors in connected health in their research work. Mohammadi et al. [42] used a supervised algorithm for stress recognition which achieved a promising accuracy of 94.4 ± 2.5%.

## 6. Conclusions and Future Works

This research paper proposes an image-encoding-based deep neural network model for the classification of mental stress of an individual. The experiment was conducted after thoroughly understanding the format and structure of the publicly available multimodal WESAD dataset as well as the SWELL dataset. While experimenting, various other related works regarding the WESAD and the SWELL datasets were also inspected, along with their training as well as testing accuracies. The proposed work introduces a new method of human stress detection using deep learning methods and in no way underestimates other efforts or related works which have been conducted with the same dataset. The proposed image-encoding-based deep neural network produces classification accuracies of 94.77% and 99.39% for WESAD and SWELL datasets, respectively, which is quite impressive. The proposed model is also found to surpass most of the previous work performed on mental stress detection. Further work will include improving the CNN model by introducing more layers.

The proposed work was performed considering only the chest data of the WESAD dataset. In the future, wrist data will be taken into consideration, and in addition the model will also be tested for other subjects whose data have been recorded both in terms of the chest and wrist. It is also being planned to apply an attention-based mechanism in the CNN model, which is currently being experimented with for a better and more promising result. The proposed work has performed better as compared to the accuracies achieved by some of the previous research works, which also introduces a new method of stress detection by plotting the bio-signals into images after collecting the data in the form of time series and extracting them properly, followed by required normalization.

For the SWELL dataset the quantity is much less as compared to the WESAD dataset, so it took a smaller number of epochs to train it properly, for which we could not plot the loss function and accuracy due to the smaller number of epochs. In the future, we intend to develop a considerably larger wearable sensor dataset mainly for huge training of our model and evaluate the performance after training. We mainly used a custom-built CNN model for this experiment, but we can also use an attention layer mechanism in this model to make the model better, thereby enhancing the overall performance. An attention layer will be used in order to focus more on some of the selected layers of the model, thereby ignoring others. With an attention mechanism, all the hidden layers will be retained and used during the decoding process. However, the experiment can also be performed with the help of other well-known image encoding methods such as Markov Transition Field and Recurrence Plot before training with the help of a deep neural network.

## Figures and Tables

**Table 1:** Overview of the customized CNN architecture used in the present work.

Layer (Type)	Activation Function	Output Shape	Parameters
Conv 2D 1	ReLU	(None,6,6,64)	640
Batch Normalization	-	(None,6,6,64)	256
Conv 2D 2	ReLU	(None,4,4,64)	36,928
Batch Normalization	-	(None,4,4,64)	256
Conv 2D 2	ReLU	(None,2,2,64)	36,928
Batch Normalization	-	(None,2,2,64)	256
Max Pooling Layer	-	(None,1,1,64)	0
Flatten	-	(None,64)	0
Dense Layer 1	-	(None,6)	390
Output Dense Layer	Softmax	(None,4)	28

**Table 2:** Performance of our proposed image-encoding-based deep neural network model for class-wise accuracy of WESAD dataset with different stress levels.

Stress Level	Accuracy	Precision	Recall	F1 Score
Meditation (0)	94.55%	0.92	0.95	0.93
Baseline (1)	95.15%	0.97	0.95	0.96
Stress (2)	97.06%	0.95	0.97	0.96
Amusement (3)	92.36%	0.95	0.92	0.94
**Average**	**94.77%**	**0.95**	**0.95**	**0.95**

**Table 3:** Performance of our proposed model for class-wise accuracy of SWELL dataset with different stress levels.

Stress Level	Accuracy	Precision	Recall	F1 Score
No Stress (0)	99.84%	0.99	1.00	1.00
Time Pressure (1)	99.20%	1.00	0.99	0.99
Interruption (2)	99.14%	1.00	0.99	0.99
**Average**	**99.39%**	**0.99**	**0.99**	**0.99**

**Table 4:** Overall performance results attained by the proposed image-encoding-based deep neural network on both WESAD and SWELL datasets.

Dataset	Training Accuracy	Testing Accuracy	F1 Score	Precision	Recall
WESAD	99.43%	94.77%	0.95	0.95	0.95
SWELL	99.50%	99.39%	0.99	0.99	0.99

**Table 5:** Comparison of our proposed image encoding-based deep neural network model with previously proposed works related to WESAD dataset.

Research Work [Ref.]	Model Used	Year of Publication	Testing Accuracy
*Stress Detection with Machine Learning and Deep Learning using Multimodal Physiological Data.* [4]	Machine learning techniques (K-Nearest Neighbour, Linear Discriminant Analysis, Random Forest, Decision Tree, AdaBoost, and Kernel Support Vector Machine)	2020	84.32%
*Stress Classification and Personalization: Getting the most out of the least.* [16]	CNN	2021	92.85%
*A New Physiology-based Objective Mental Stress Detection Technique with Reduced Feature Set and Class Imbalanced Dataset Management.* [17]	Machine learning techniques (Random Forest Classifier, Randomized Tree (ERT))	2021	97.08%
*MoStress: a Sequence Model for Stress Classification.* [14]	RNN	2022	86%
*Semi-Supervised Generative Adversarial Network for Stress Detection Using Partially Labeled Physiological Data.* [43]	Semi-supervised learning (SSL) model	2022	90.31%
*Evaluating different configurations of machine learning models and their transfer learning capabilities for stress detection using heart rate* [44]	Artificial Intelligence (AI) models, Supervised Multi-Layer Perceptron (MLP)	2022	88.89%
**Proposed work**	**CNN using GAF images**	**2022**	**94.8%**

**Table 6:** Comparison of our proposed image encoding-based deep neural network model with previously proposed works related to SWELL dataset.

Research Work	Model Used	Year of Publication	Testing Accuracy
*Stress Detection Using Machine Learning Classifiers in Internet of Things Environment* [19]	Machine learning methods along with IoT and cloud computing	2019	98%
*Bayesian active learning for wearable stress and affect detection* [20]	Bayesian neural network technique using Monte-Carlo Dropout	2020	90.38%
*Employing Multimodal Machine Learning for Stress Detection* [35]	Multimodal framework based on AI	2021	96.09%
**Proposed work**	**CNN using GAF images**	**2022**	**99.39%**

## Data Availability

No new data were created or analysed in this study. Data sharing is not applicable to this article. We used only publicly available datasets for experimentation.

## Code Availability Statement

The source codes related to the present work can be found at: https://github.com/sayang14/Classification-of-Mental-Stress-from-Wearable-Physiological-Sensors-Using-Deep-Neural-Network (accessed on 9 October 2022).
